# Pharmacogenetics and antidepressant treatment outcomes in pregnancy: a Danish-population based study

**DOI:** 10.1192/j.eurpsy.2022.297

**Published:** 2022-09-01

**Authors:** M. Mushtaq, K. Ishtiak-Ahmed, J. Thirstrup, C. Gasse

**Affiliations:** 1 Aarhus University Hospital Psychiatry, Department Of Affective Disorders, Aarhus N, Denmark; 2 Aarhus University, Department Of Clinical Medicine, Aarhus N, Denmark

**Keywords:** Depression, Antidepressants, pharmacogenetics, Pregnancy

## Abstract

**Introduction:**

Depression in pregnancy is common and often requires treatment with antidepressant drugs. Most antidepressants are metabolized by the cytochrome P450 system (CYP), in particular CYP2C19 and -2D6, both of which are genetically polymorphic. Additionally, the activity of these enzymes is altered during pregnancy.

**Objectives:**

To investigate pharmacogenetic variability regarding CYP2C19 and -2D6 in pregnant users of antidepressants and treatment outcomes.

**Methods:**

The study population comprises all women born between 1981-1999, who gave birth to at least one child before December 2015 identified from the large Danish population-based iPSYCH2012 case-cohort study sample linked to information on genetic variants, prescription drug use and outcome data. Pharmacogenetic genotypes and phenotypes of CYP2C19 and CYP2D6 will be categorized into poor, (PM), intermediate, (IM), extensive, (EM), rapid (RM) and ultra-rapid metabolizers (RM) using array-based SNP information. Antidepressant drug use and comedication during pregnancy will be assessed based on prescription data. Outcomes include treatment discontinuation, switching and psychiatric hospitalizations. Cox regression analysis will be performed to estimate the hazard ratios comparing the rates of the different outcomes in people with different phenotypes, compared with EM adjusted for a number of confounding factors.

**Results:**

Based on previous research we will be able to identify approximately 6531 pregnant women with a psychiatric history. Among those, we estimate to find 14 PM, 161 IM, 285 EM, 168 RM and 25 UM of CYP2C19, and 27 PM, 218 IM and 408 EM of CYP2D6. Exposure to antidepressants is estimated at 10%.

**Conclusions:**

We expect to be able to present the results at the conference.

**Disclosure:**

No significant relationships.

